# A turn-on fluorescence sensor for the highly selective detection of Al^3+^ based on diarylethene and its application on test strips†

**DOI:** 10.1039/c9ra00716d

**Published:** 2019-04-02

**Authors:** Junfei Lv, Yinglong Fu, Gang Liu, Congbin Fan, Shouzhi Pu

**Affiliations:** Jiangxi Key Laboratory of Organic Chemistry, Jiangxi Science and Technology Normal University Nanchang 330013 PR China congbinfan@163.com pushouzhi@tsinghua.org.cn +86 791 83805212 +86 791 83805212 +86 791 83831996

## Abstract

A novel turn-on fluorescent sensor for Al^3+^ based on photochromic diarylethene with a 2-hydroxybenzhydrazide unit has been successfully designed and synthesized. The photochromic and fluorescent characteristics were studied methodically in methanol under irradiation using UV/vis light and induced by Al^3+^/EDTA. This fluorescent sensor was highly selective toward Al^3+^ with an obvious fluorescent color change from dark blue to blue. The Job's plot and mass spectrometry (MS) analysis indicate a binding stoichiometry of 1 : 1 between the fluorescent sensor and Al^3+^. Moreover, a test strip containing this fluorescent sensor was prepared to allow for the easy detection of Al^3+^ in water. Finally, a logic circuit was designed using four input signals (In1: UV; In2: vis; In3: Al^3+^; In4: EDTA) and one output signal.

## Abstract 2

A novel fluorescent sensor, 1O, based on photochromic diarylethene with a 2-hydroxybenzhydrazide unit was designed and synthesized and can be used to recognize Al^3+^ in methanol (2.0 × 10^−5^ mol L^−1^).

## Graphical abstract

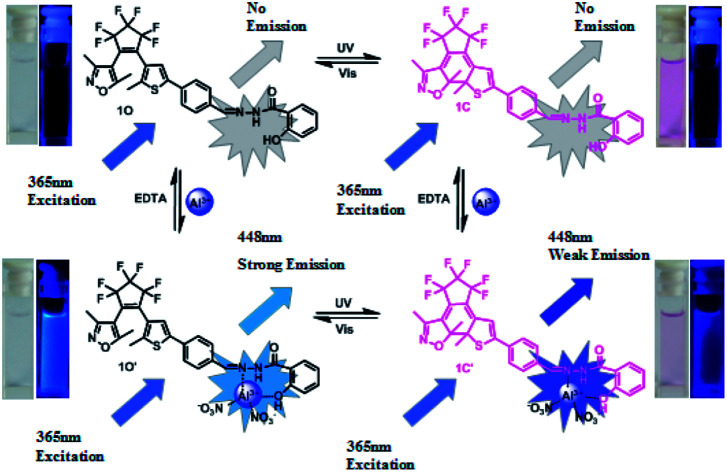

## 1. Introduction

Aluminum is the most abundant metal element in the earth's crust, most of it exists as a compound, such as aluminum oxide, aluminum hydroxide, and potassium sulfate and a significant amount exists as Al^3+^ in natural waters and in many biological tissues.^1,2^ Furthermore, aluminum plays a very important role in the daily life of humans,^3,4^ for example, aluminum products are widely used as food additives, cooking utensils, aluminum-based pharmaceuticals, in the automotive and aeronautic transport industry, and so forth.^5^ With increased research, more and more studies have confirmed that excess Al^3+^ is quite toxic to biological systems,^6^ for example, Al^3+^ toxicity may be related to Alzheimer's and Parkinson's diseases.^7,8^ On the one hand, there is an increased risk of a large amount of free Al^3+^ being released because of acid rain dropping onto the soil and causing the release of aluminum from the soil. It is believed that 40% of acidic soil in the world contains a high concentration of Al^3+^, which influences plant growth.^9,10^ On the other hand, this could lead to an increase of Al^3+^ in our lives because of the widespread use of aluminum compounds in water treatment, cooking utensils, food additives and so forth. Al^3+^ can spread to the tissues of humans and animals and eventually accumulates in the bones.^11,12^ A high concentration of Al^3+^ gives rise to serious bone diseases, such as myopathy, microcytic hypochromic anemia, dialysis dementia, encephalopathy, neuronal myopathy and can even lead to central nervous system damage.^13,14^ The World Health Organization recommends a daily intake of approximately 3–10 mg.^15,16^ Therefore, it is important to be able to detect the amount of Al^3+^ in the environment owing to the risks to human health. To date, many conventional methods have been used to detect Al^3+^, such as atomic absorption spectroscopy^17^ and inductively coupled plasma emission spectroscopy^18^ for example. However, they require expensive instruments, complicated operating procedures, and high operating costs. Compared with the traditional methods, fluorescent chemical sensors have many advantages, such as a simple operation, low cost and high sensitivity and they have attracted wide ranging attention.^19–21^

In the past few years, large numbers of photoresponsive compounds have been reported for the detection of ions.^22–25^ Among the photoresponsive materials that have been reported, photochromic diarylethenes are considered to be the most promising photo-switchable molecules on account of their prominent thermal stability, excellent fatigue resistance, and rapid response.^26–28^ Diarylethene compounds can be functionalized as fluorescence sensors as their fluorescence can be reversibly adjusted by alternating ultraviolet light and visible light. In addition, it is well-known that 2-hydroxybenzhydrazide can react with the aldehyde group of diarylethene to form a Schiff base group, which is one of the most attractive and effective functional groups owing to their easy preparation and affluent bonding sites (N and O atoms).^29–33^ Taking consideration of these aspects, we have designed and synthesized a diarylethene derivative 1O, as a target fluorescent sensor that can forcefully bind metal ions through the introduction of a Schiff base moiety.^34–36^

**Scheme 1:**
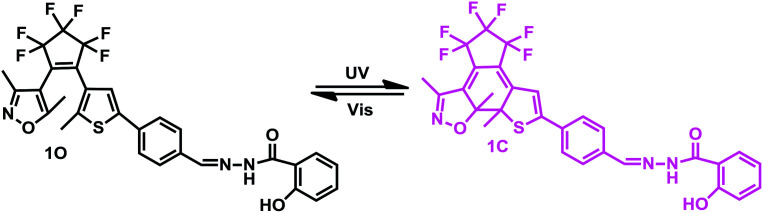
Photochromism of diarylethene 1O.

Therefore, a new diarylethene derivative 1O, containing a 2-hydroxybenzhydrazide Schiff base unit was designed and synthesized as a fluorescent sensor for detecting Al^3+^ with a high selective and with sensitive characteristics. The structure of 1O was characterized using ^1^H NMR, ^13^C NMR, and infrared spectroscopy (IR), and the results are displayed in the ESI (Fig. S1–S3†). The multifunctional fluorescent switching characteristics induced by Al^3+^/EDTA and UV/vis light were systematically investigated. The photochromism of the diarylethene is shown in Scheme 1 and the analytical performance for the detection of Al^3+^ is compared with other reported sensors in Table 1. Compared with these reported fluorescent sensors, the diarylethenes fluorescent sensor (1O) has a specific recognition ability for Al^3+^ and the sensing process is not affected by interference from other metal ions. In addition, 1O exhibited multi-control fluorescence switching behaviors in the presence of Al^3+^ and lights. Moreover, sensor 1O has been successfully applied as a test paper and for the construction of a logic gate.

**Table 1:** Comparative study of the analytical performance of 1O with other reported sensors

Compound number	Structure	Interferents	*K*_a_ (M^−1^)	Test strip	Logic circuit	Ref.
1O	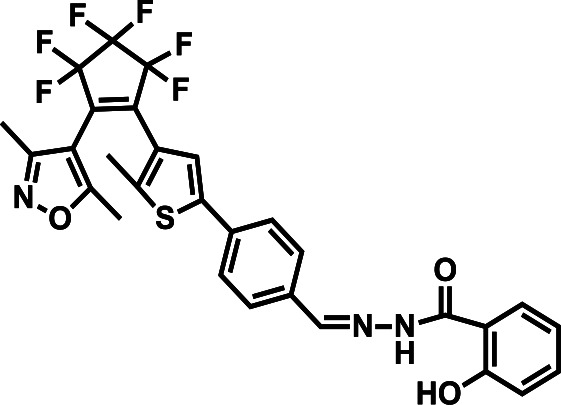	None	4.72 × 10^4^	Yes	Yes	This work
2	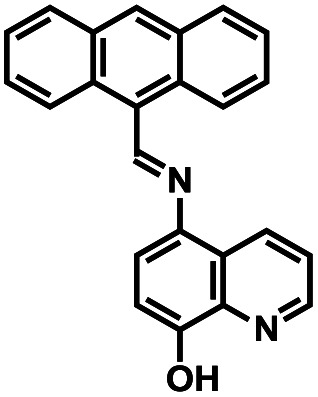	Pb^2+^	2.10 × 10^2^	No	No	37
3	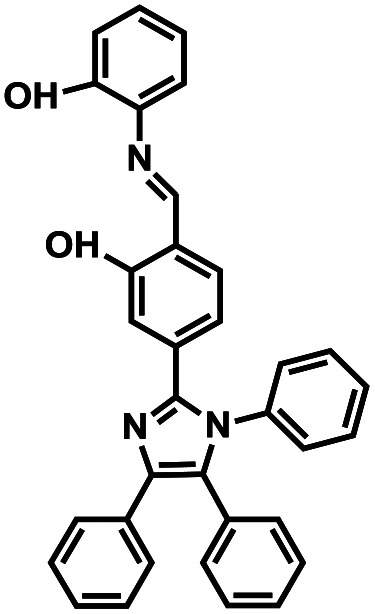	Hg^2+^, Fe^3+^	8.46 × 10^5^	Yes	No	38
4	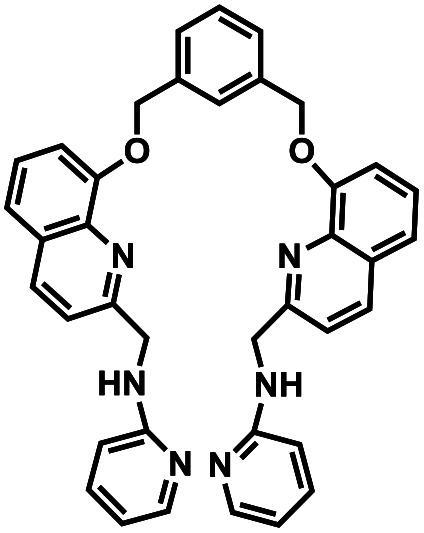	Cr^3+^	2.11 × 10^3^	No	No	39
5	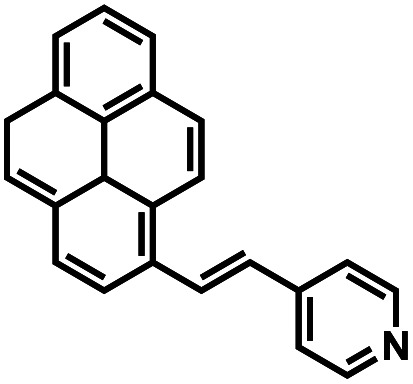	Hg^2+^	1.00 × 10^9.08^	Yes	No	40
6	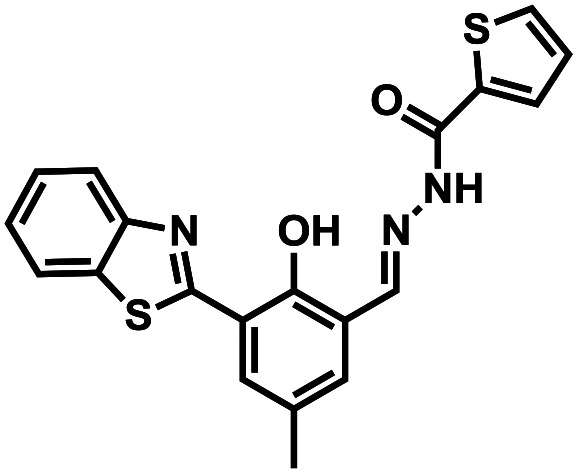	Zn^2+^	—	Yes	No	41

## 2. Experiments

### 2.1 General procedures and materials

All solvents used were of analytical grade and were used without further purification. However, the solvents used in the characterization test were of spectroscopic grade. The metal ions Zn^2+^, Cd^2+^, Fe^3+^, Pb^2+^, Ca^2+^, Co^2+^, Cr^3+^, Ni^2+^, Mg^2+^, Sr^2+^, Al^3+^, Cu^2+^ were all dissolved in 10 mL of deionized water using their respective nitrates (0.1 mmol). The counter-ions of Ba^2+^, Mn^2+^, Hg^2+^, K^+^, are chloride ions, therefore these were used instead. ^1^H NMR and ^13^C NMR spectra were recorded using CDCl_3_ and DMSO-d_6_ as the solvents on a Bruker AV400 (400 MHz) spectrometer with tetramethylsilane (TMS) as the internal standard. The melting points were obtained using a WRS-1B melting point apparatus. Mass spectra were measured on an AB SCIEX Triple TOFTM 4600 instrument. IR were collected on a Bruker Vertex-70 spectrometer. Elemental analysis was carried out using a PE CHN 2400 analyzer. The fluorescence quantum yield was recorded using an absolute PL quantum yield spectrometer QY. Fluorescence spectra were measured on a Hitachi F-4600 fluorescence spectrophotometer. The UV-vis absorption spectra were measured with an Agilent 8453 UV-vis spectrometer. Light irradiation experiments were conducted using a SHG-200 UV lamp, a Cx-21 UV fluorescence analysis box and BMH-250 visible light.

### 2.2 Synthesis of 1O

**Scheme 2:**
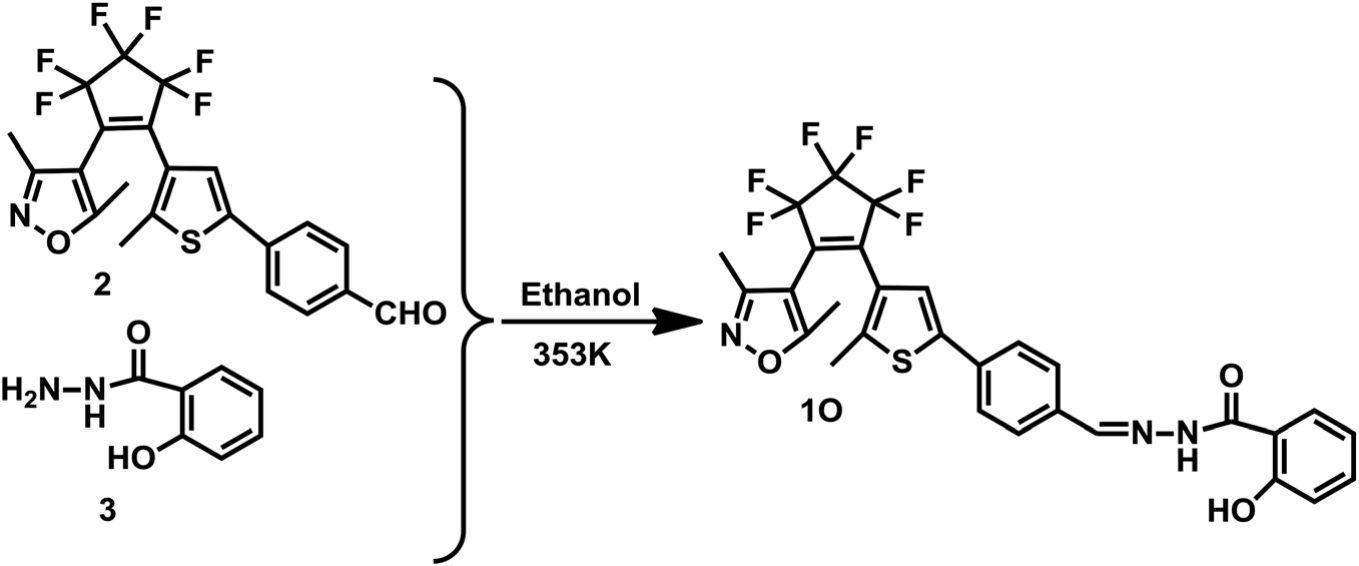
Synthetic route to diarylethene 1O.

The synthetic route to the diarylethene (1O) is shown in Scheme 2. Compound 2 was prepared using the method previously reported in the literature.^42^ The experimental characterization data and details of the procedures used to prepare 1O are detailed below.

In a 50 mL flask, compound 2 (0.49 g, 1.03 mmol) and compound 3 (0.18 g, 1.20 mmol) were dissolved in absolute ethanol (10.0 mL) and refluxed for 5 h, then cooled to room temperature and concentrated under reduced pressure. The resulting solid was recrystallized from ethanol to obtain 1O (0.36 g, 0.62 mmol) as a pale yellow solid in a 60% yield. Mp 460–461 K; ^1^H NMR (CDCl_3_, 400 MHz), *δ*(ppm): 2.01 (s, 3H), 2.13 (s, 3H), 2.28 (s, 3H), 6.95–7.01 (m, 2H, *J* = 8.0 Hz), 7.45 (t, 1H, *J* = 8.0 Hz), 7.64 (s, 1H), 7.75–7.77 (m, 2H, *J* = 8.0 Hz), 7.79–7.82 (m, 2H, *J* = 8.0 Hz), 7.90 (d, 1H, *J* = 8.0 Hz), 8.47 (s, 1H), 11.63 (d, 2H, *J* = 8.0 Hz). ^13^C NMR (DMSO-d_6_, 100 MHz): 10.7, 12.2, 14.5, 104.4, 116.3, 117.7, 117.8, 119.5, 119.9, 121.8, 124.5, 124.6, 126.1, 129.1, 129.2, 134.4, 140.9, 142.3, 148.2, 158.0, 158.6, 159.4, 165.0, 170.7. IR (KBr, *ν*, cm^−1^): 3254 (–OH), 1636 (–C=O), 1606 (–CH=N). Anal. calcd for C_29_H_21_F_6_N_3_O_3_S (%): C, 57.52; H, 3.50; N, 6.94. Found: C, 57.51; H, 3.51; N, 6.93. MS-ESI (*m/z*): 604.2 [1O–H]^−^ (calcd 604.1).

## 3. Results and discussion

### 3.1 Photochromic and fluorescent properties of 1O

**Fig. 1:**
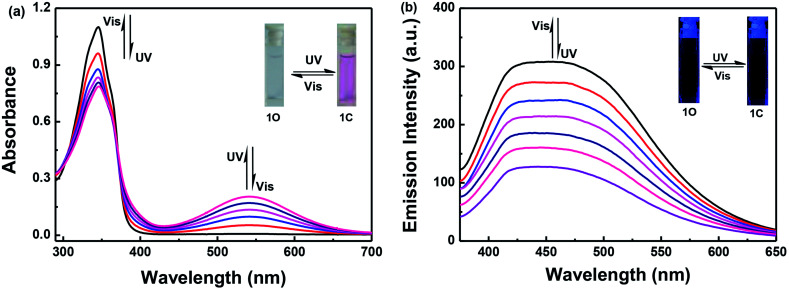
The absorption spectra and fluorescence changes of 1O with UV/vis irradiation in methanol (2.0 × 10^−5^ mol L^−1^): (a) absorption spectra and color changes; and (b) fluorescence changes when excited at 365 nm.

The photochromic and fluorescence properties of 1O were measured in methanol (2.0 × 10^−5^ mol L^−1^). As shown in Fig. 1a, the maximum absorption of 1O was observed at 346 nm (*ε*_max_ = 5.5 × 10^4^ L mol^−1^ cm^−1^) in methanol, which resulted from a π–π* transition,^43^ at the same time the solution was colorless. When irradiated with 297 nm UV light, the absorption band at 346 nm decreased and a new absorption band centered at 541 nm appeared. This was accompanied by the solution changing from colorless to purple, owing to the formation of the closed-ring isomer 1C. Conversely, when irradiated with visible light (*λ* > 500 nm), the absorption spectra of the closed-ring state 1C returns completely to the initial state 1O, and the solution changes from purple to colorless at the same time.

When excited at 365 nm light, the fluorescence emission peak of 1O appeared at 452 nm in methanol. The absolute fluorescence quantum yield of 1O was determined to be 0.004. Upon irradiating with 297 nm UV light, the emission intensity decreased, owing to the occurrence of the photocyclization reaction and the generation of the closed-ring isomer of 1C. When the photostationary state (PSS) was reached the fluorescence intensity of 1O was quenched to *ca.* 59% in methanol. The residual fluorescence cannot be decreased any further due to the incomplete cyclization reaction and the existence of the open-ring isomers which have a parallel conformation.^44^ At this time, the fluorescence emission cannot be observed by the naked eye. By using visible light (*λ* > 500 nm) to irradiate the solution of 1C, the fluorescence intensity was fully restored to that of the open-ring 1O (Fig. 1b).

### 3.2 Absorption spectrum changes induced by Al^3+^/EDTA and UV/vis light

**Fig. 2:**
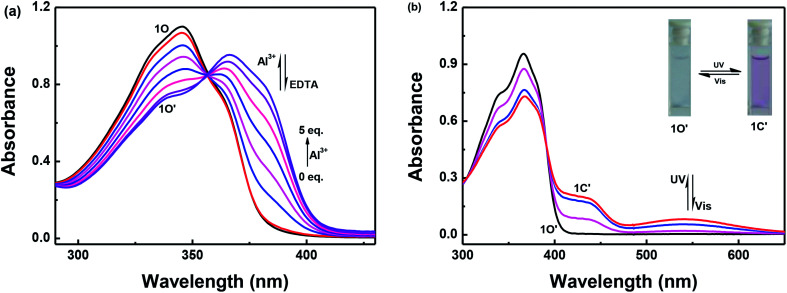
Absorption spectra changes of 1O induced by Al^3+^/EDTA and UV/vis light in methanol solution (2.0 × 10^−5^ mol L^−1^): (a) 1O induced using Al^3+^/EDTA; and (b) 1O′ induced using UV/vis light.

Fig. 2 shows the absorbance spectra changes of 1O induced by Al^3+^/EDTA and UV/vis light in a methanol solution (2.0 × 10^−5^ mol L^−1^). As shown in Fig. 2a, when 5.0 equivalents of Al^3+^ (0.1 mol L^−1^) was gradually added to the methanol solution of 1O, the maximum absorption peak at 345 nm decreased, the absorbance spectra gradually red-shifted and a new absorption band appeared that was centered at 366 nm owing to the formation of the 1O–Al^3+^ (1O′) complexes. Upon addition of Al^3+^ to the solution of 1O, the color of the solution did not change significantly.

As shown in Fig. 2b, 1O′ also underwent photochromism under UV/vis light irradiation. When irradiated with UV light at 297 nm, the color of the solution of 1O′ changed from colorless to purple and a new absorption band centered at 542 nm emerged owing to the formation of the closed-ring isomer 1C′. Similarly, under visible light (*λ* > 500 nm) irradiation, the absorption band centered at 542 nm disappeared completely and the system returned to the 1O′ state, the color of the solution changed from purple to colorless. The maximum absorption peak of 1C was observed at 347 nm. When Al^3+^ was added to the solution of 1C, the absorption peak at 347 nm red-shifted to 368 nm and increased slightly. At the same time, the color of the solution changed from purple to light purple owing to the formation of 1C′. The absorption spectra returned to the initial state of 1C when as excess of EDTA (0.1 mol L^−1^) was added to the solution of 1C′ (Fig. S5†). This indicated that a reversible transformation between 1C and 1C′ could be induced using Al^3+^ and EDTA.

### 3.3 Fluorescence response to metal ions

**Fig. 3:**
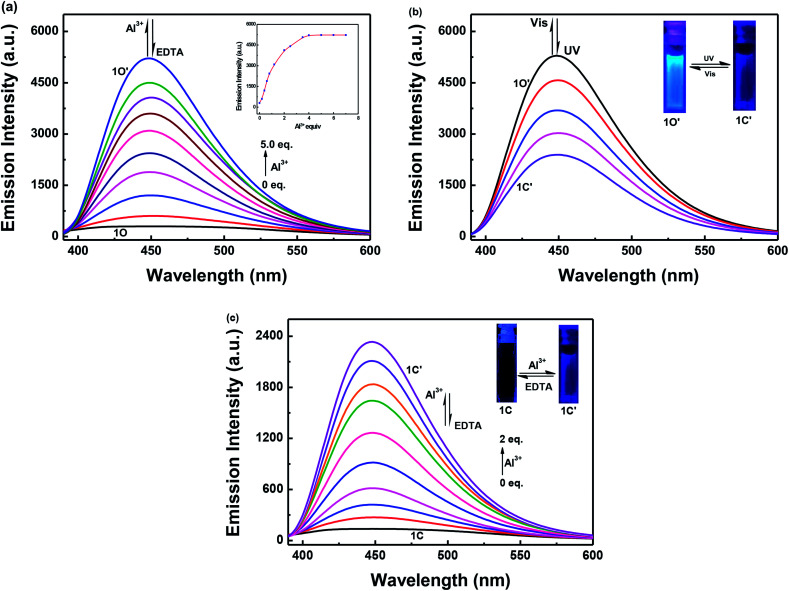
Fluorescence intensity changes induced by Al^3+^/EDTA and UV/vis light in methanol solution (2.0 × 10^−5^ mol L^−1^): (a) 1O induced using Al^3+^/EDTA; (b) 1O′ induced using UV/vis light; and (c) 1C induced using Al^3+^/EDTA.

The fluorescence intensity of 1O in a methanol solution (2.0 × 10^−5^ mol L^−1^) changes following induction using Al^3+^/EDTA and UV/vis light. As shown in Fig. 3a, the emission intensity of 1O at 448 nm gradually increased when Al^3+^ was increased from 0 to 5.0 equivalents, followed by a plateau upon further addition. At this time, a new compound 1O′, was formed, the fluorescence color of the solution changed from dark blue to blue. Compared with 1O, the fluorescence of 1O′ was enhanced by 18-fold at the plateau. The absolute fluorescence quantum yield of 1O′ was determined to be 0.029. The fluorescence intensity of 1O was restored when excess EDTA was gradually added. This is due to the occurrence of a complexation–dissociation reaction between Al^3+^ and EDTA. Under UV irradiation at 297 nm, the fluorescence intensity of 1O′ dramatically declined with a fluorescence color change from blue to dark blue owing to the formation of the closed-ring isomer 1C′, and the fluorescent relative intensity decreased from 5289 to 2393. Moreover, the emission intensity of 1C′ returned to that of 1O′ upon irradiation with an appropriate wavelength of visible light (*λ* > 500 nm) (Fig. 3b). As shown in Fig. 3c, a fluorescence titration of 1C using Al^3+^ was performed in methanol. When 2.0 equivalents of Al^3+^ was added to 1C, the emission intensity reached the maximum value at 448 nm. Compared with 1C, the fluorescence of 1C′ was enhanced 17-fold at the plateau. When the excess EDTA was added, the fluorescence intensity returned to the initial state of 1C. It was shown that 1C and 1C′ could be transformed into each other.

**Fig. 4:**
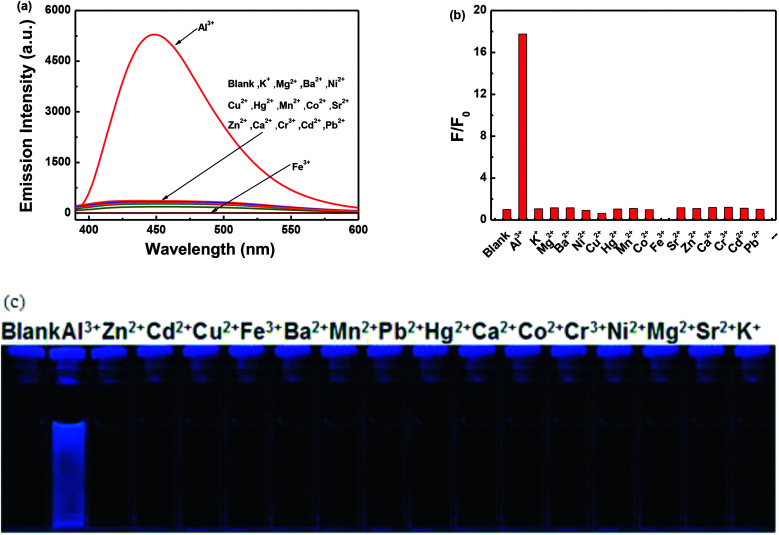
Changes in the fluorescence of 1O induced by the addition of various metal ions (10.0 equiv.) in methanol (2.0 × 10^−5^ mol L^−1^): (a) emission spectral changes; (b) emission intensity changes; and (c) photograph of the change in fluorescence.

The fluorescence response of 1O, which was induced by the addition of different metal ions such as Al^3+^, K^+^, Ca^2+^, Hg^2+^, Sr^2+^, Cd^2+^, Zn^2+^, Mg^2+^, Mn^2+^, Ba^2+^, Ni^2+^, Co^2+^, Pb^2+^, Sn^2+^, Cu^2+^, Cr^3+^, and Fe^3+^ is shown in Fig. 4. It can be seen that the fluorescence spectra of 1O was not obviously changed, except for the addition of Al^3+^. As shown in Fig. 4a, when various metal ions (4.0 μL, 0.1 mol L^−1^) were added to the methanol solution (2.0 × 10^−5^ mol L^−1^) containing 1O, only Al^3+^ caused a drastic fluorescence enhancement at 449 nm. At the same time, the fluorescent color of 1O changed from dark blue to blue (Fig. 4c). The increase in the fluorescence intensity could be attributed to the chelating enhanced fluorescence (CHEF). In addition, the stable chelation of 1O with Al^3+^ inhibited the isomerization of C=N.^45,46^ As shown in Fig. 4b, the fluorescence intensity of Al^3+^ was much higher than that of the other metal ions. Thus, 1O could be used as a highly selective fluorescent sensor for Al^3+^ recognition.

**Fig. 5:**
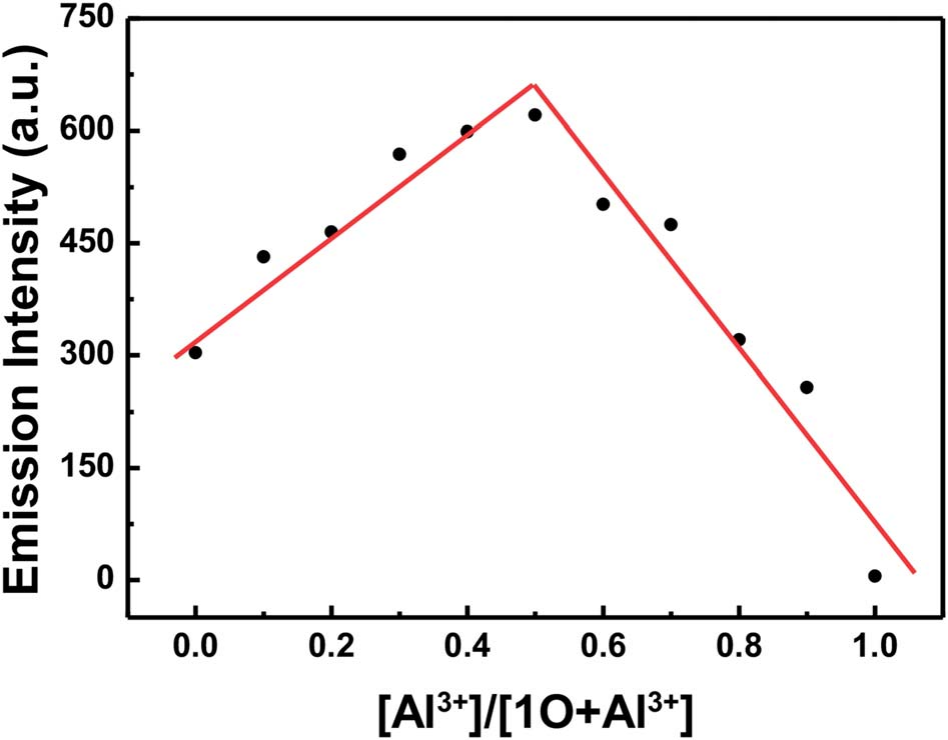
Job's plot showing the 1 : 1 complex of 1O and Al^3+^.

In order to calculate the binding ratio between 1O and Al^3+^, a Job's plot was performed by fluorescence titration according to the method previously reported.^47^ It can easily be seen that the emission intensity of complexes 1O–Al^3+^ approached the maximum value when the molar fraction of [1O]/([1O] + [Al^3+^]) was about 0.5, indicating that 1O was bound to Al^3+^ with a binding stoichiometry of 1 : 1 (Fig. 5).

**Fig. 6:**
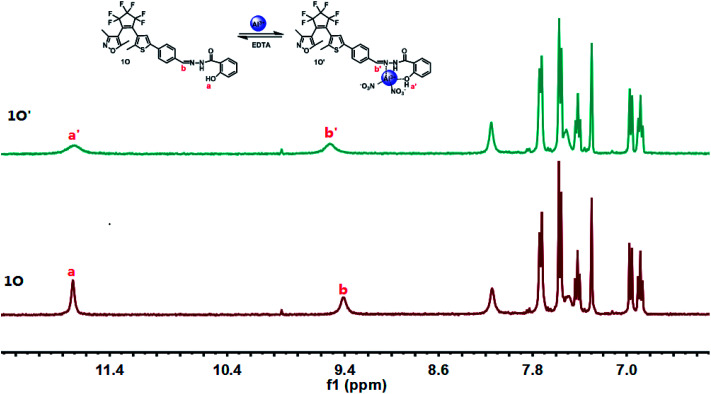
Changes in the ^1^H NMR spectra of 1O and 1O′ in CD_2_Cl_2_ (inset shows the proposed binding mode of the 1O′ complex).

A ^1^H NMR titration experiment was performed in CD_2_Cl_2_ to further investigate the binding mode between 1O and Al^3+^. As shown in Fig. 6, the signal peak Ha at 11.71 ppm belongs to the protons of the hydroxyl (–OH), and the signal peak Hb at 9.41 ppm corresponds to the protons of the Schiff unit (–CH=N). With the addition of Al^3+^, the signal peak Ha became wider and weaker, indicating that a bond between the hydroxyl group and Al^3+^ was formed. In addition, the signal peak Hb shifted from 9.41 to 9.52 ppm, showing the formation of a bond between the Schiff unit (–CH=N) and Al^3+^. The above results indicated that the O of the hydroxyl group and the N(–CH=N) on the Schiff base are the optimal binding sites. To further confirm the binding mechanism of 1O and Al^3+^, electrospray ionization-mass spectrometry (ESI-MS) experiments were performed as shown in Fig. S4.† An ESI-MS peak for 1O at 604.2 *m*/*z* was observed and assigned to [1O–H^+^]^−^ (calcd 604.1). When excess amounts of Al^3+^ were added, a new ESI-MS peak at 754.0 *m*/*z* was observed due to the formation of complexes, this was assigned to [1O + Al^3+^ + 2NO_3_^−^ − 2H]^−^ (calcd 754.1). This result further confirmed that the formation of a 1 : 1 complex between 1O and Al^3+^. In addition, the association constant (*K*_a_) for the complexation of 1O with Al^3+^ was calculated to be 4.72 × 10^4^ L mol^−1^ (*R* = 0.9913) using the Hildebrand–Benesi equation^48^ (Fig. S6†). According to the reported method,^49^ the detection limit (LOD) of 1O for Al^3+^was calculated to be 1.24 × 10^−5^ mol L^−1^ (Fig. S7†).

**Fig. 7:**
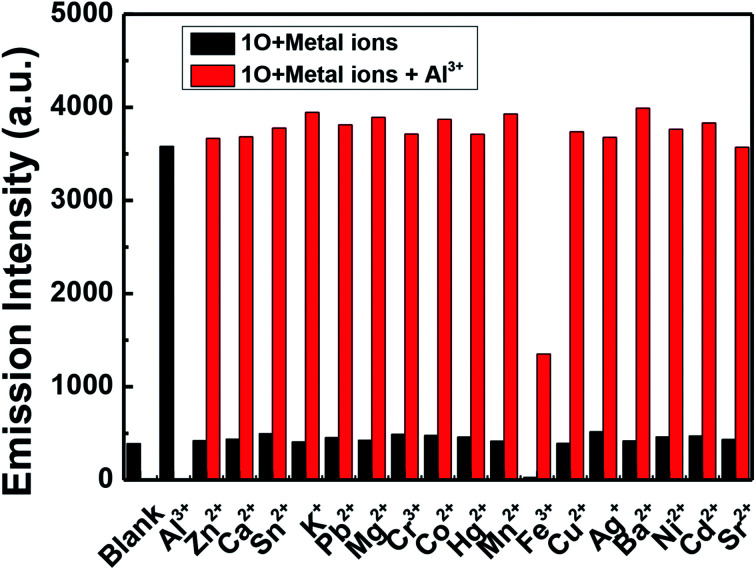
Competitive tests showing the fluorescence response of 1O to various metal ions (10.0 equiv.) in methanol (2.0 × 10^−5^ mol L^−1^). Black bars: 1O with different metal ions; red bars: 1O with different competing metal ions and Al^3+^.

In order to further determine the selectivity of 1O to Al^3+^ in methanol solution, competitive experiments were performed. As shown in Fig. 7, the fluorescence intensity showed no obvious changes upon adding Al^3+^ (10.0 equiv.) to a solution of 1O in the presence of various metal ions (10.0 equiv.) except for Fe^3+^, indicating that 1O has a good selectivity for Al^3+^.

### 3.4 Application on test strips

**Fig. 8:**
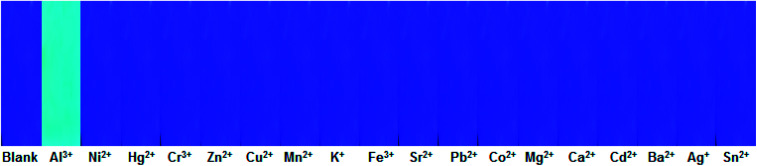
Photograph showing the change in the fluorescence color of 1O on test strips immersed in aqueous solutions of various metal ions (0.1 mol L^−1^).

In order to make testing of Al^3+^ more convenient on site, a test strip coated with 1O was made. In this experiment, a Whatman filter paper was immersed in a methanol solution (1.0 × 10^−3^ mol L^−1^) of 1O and dried at room temperature. Then, the drying test strip was immersed in a solution of various metal ions, and the fluorescence color of the test strip was observed under UV light. As shown in Fig. 8, only the test strip immersed in the Al^3+^ solution showed a distinct color change (strong fluorescence emission). Moreover, an increase in the Al^3+^ concentration gave a more pronounced fluorescence effect (Fig. S8†). Therefore, this method can be used to detect Al^3+^ more conveniently.

### 3.5 Application in logic circuits

**Fig. 9:**
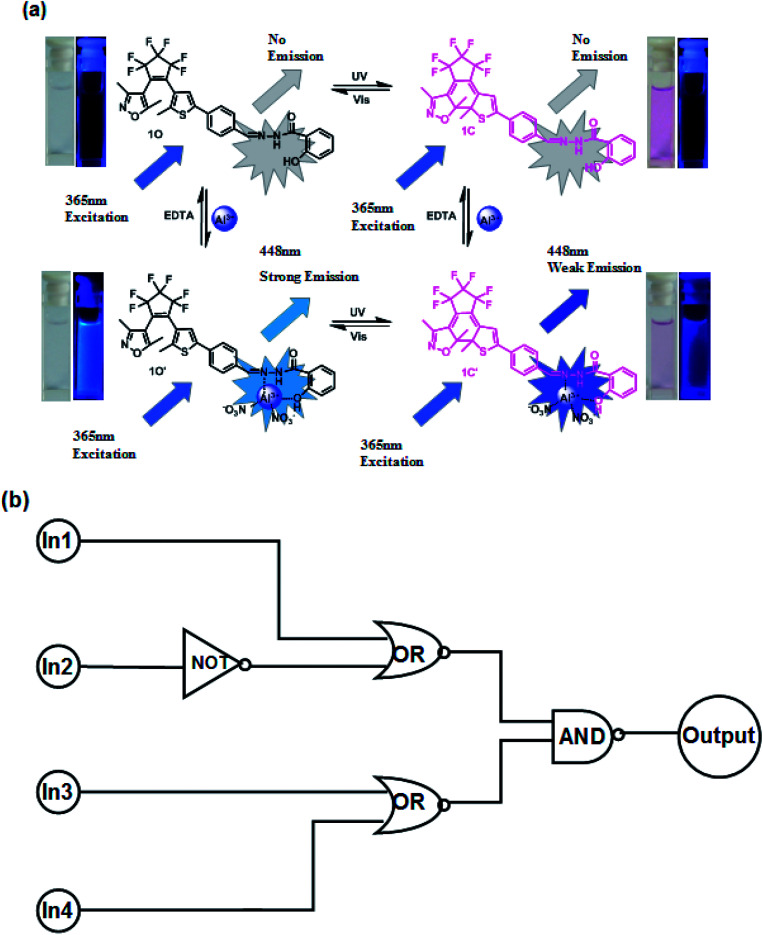
(a) Photochromism, structures, and fluorescence changes of 1O induced by Al^3+^/DETA and UV/vis light. (b) Combinational logic circuit equivalent to the truth table given in Table 2: In1 (UV); In2 (vis); In3 (Al^3+^); and In4 (EDTA).

As described above, the photochromic properties of diarylethene 1O could be effectively modulated by stimulation with UV/vis light, and Al^3+^/EDTA. The dual-controlled photoswitching behavior of diarylethene 1O is shown in Fig. 9a. Based on these characteristics, a combinational logic circuit consisting of four input signals and one output signal had been constructed, the input signals included In1 (297 nm UV light), In2 (*λ* > 500 nm visible light), In3 (Al^3+^), and In4 (EDTA) and the output signal was a change in the fluorescence intensity at 448 nm (Fig. 9b). The input signal in the logic circuit was ‘on’ or ‘off’, corresponding to the different Boolean values of ‘1’ or ‘0’. The emission intensity of diarylethene at 452 nm was considered to be the original value, and the fluorescence intensity of 1O was significantly enhanced after the addition of Al^3+^. When the emission intensity at 448 nm was 18 times greater than the original value, the output signal could be regarded as an ‘on’ state with a Boolean value of ‘1’; otherwise, it was treated as an ‘off’ state with a Boolean value of ‘0’. Under the stimulation of different conditions, diarylethene 1O demonstrated an on–off–on fluorescence switching behavior. Therefore, each input from the four strings would give a corresponding output signal. For example, if the input string is ‘1, 0, 0 and 0’, the corresponding input signals ln1, ln2, ln3 and ln4 are ‘on, off, off and off’ respectively, under these conditions, diarylethene 1O would be converted to 1C by stimulating with 297 nm light, and the fluorescence emission intensity would be reduced. As a result, the corresponding output signal was ‘off’, and the output digit was ‘0’. Similarly, the same on–off–on signal would occur under the stimulation of the other conditions of fluorescence switching and this combinational logic circuit was composed of all possible logical strings, as shown in Table 2.

**Table 2:** Truth table for all possible strings of four binary-input data and the corresponding output digit

Input				Output
In1 (UV)	In2 (vis)	In3 (Al^3+^)	In4 (EDTA)	
0	0	0	0	0
1	0	0	0	0
0	1	0	0	0
0	0	1	0	1
0	0	0	1	0
1	1	0	0	0
1	0	1	0	0
1	0	0	1	0
0	1	1	0	1
0	1	0	1	0
0	0	1	1	0
1	1	1	0	1
1	1	0	1	0
1	0	1	1	0
0	1	1	1	0
1	1	1	1	0

## 4. Conclusions

In conclusion, a novel diarylethene derivative with a 2-hydroxybenzoic acid hydrazide unit was designed and synthesized, and showed high selectivity and sensitivity for Al^3+^ in methanol solution. It showed multiple responses when induced using UV/vis light and Al^3+^/EDTA. When Al^3+^ was added to a methanol solution of 1O, the fluorescent color changed from dark blue to blue. This diarylethene derivative could be used as a fluorescent sensor to “recognize” Al^3+^. Moreover, a test strip with a sensor function was successfully prepared and a logic circuit was constructed on the basis of the unimolecular platform. This work provides a useful strategy for the development of chemical sensors, the monitoring of Al^3+^ in the environment and the potential applications of molecular logic circuits.

## Conflicts of interest

There are no conflicts to declare.

## Supplementary Material

RA-009-C9RA00716D-s001
