# Supplementary material for: A turn-on fluorescence sensor for the highly selective detection of Al3+ based on diarylethene and its application on test strips
Source: RSC Adv. 2019 Apr 2;9(18):10395–404. doi: 10.1039/c9ra00716d (PMC9062525; doi:10.1039/c9ra00716d)
Supplement: RA-009-C9RA00716D-s001 [file RA-009-C9RA00716D-s001.pdf]

Electronic Supplementary Material (ESI) for RSC Advances.
This journal is © The Royal Society of Chemistry 2019

Supporting Information

A turn-on fluorescence sensor for highly selective detection of Al^{3+} based on diarylethene and its application by test strips

Junfei Lv, Yinglong Fu, Gang, Liu, Congbin Fan* and Shouzhi Pu*

Jiangxi Key Laboratory of Organic Chemistry, Jiangxi Science and Technology Normal University, Nanchang 330013, PR China

* *Corresponding authors.* Tel./fax: +0791 83805212 (C. Fan), +86 791 83831996 (S. Pu).

E-*mail:* congbinfan@163.com (C. Fan), pushouzhi@tsinghua.org.cn (S. Pu).

Contents

Figure S1. ^{1}H NMR spectrum of **1O**.

Figure S2. ^{13}C NMR spectrum of **1O**.

Figure S3. IR spectra of **1O**.

Figure S4. MS-ESI spectrum of **1O**.

Figure S5. Absorption spectra changes of **1C** induced by Al^{3+}/EDTA in methanol solution (2.0×10^{-5} mol L^{-1}).

Figure S6. The binding constant of **1O** with Al^{3+} was calculated to be 4.72×10^{4} L·mol^{-1}.

Figure S7. The limit of detection (LOD) for Al^{3+} was 1.24×10^{-5} mol L^{-1}.

Figure S8. Fluorescence photos of Al^{3+} at different concentrations.

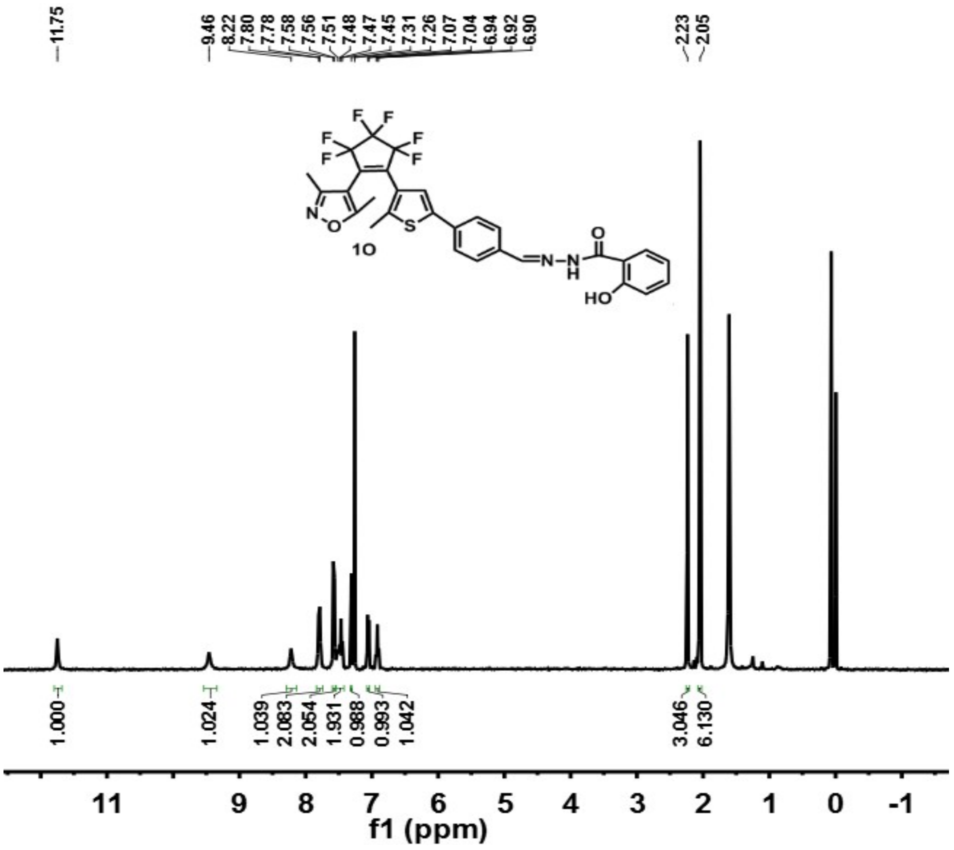

Figure S1. ^{1}H NMR spectrum of **1O**.

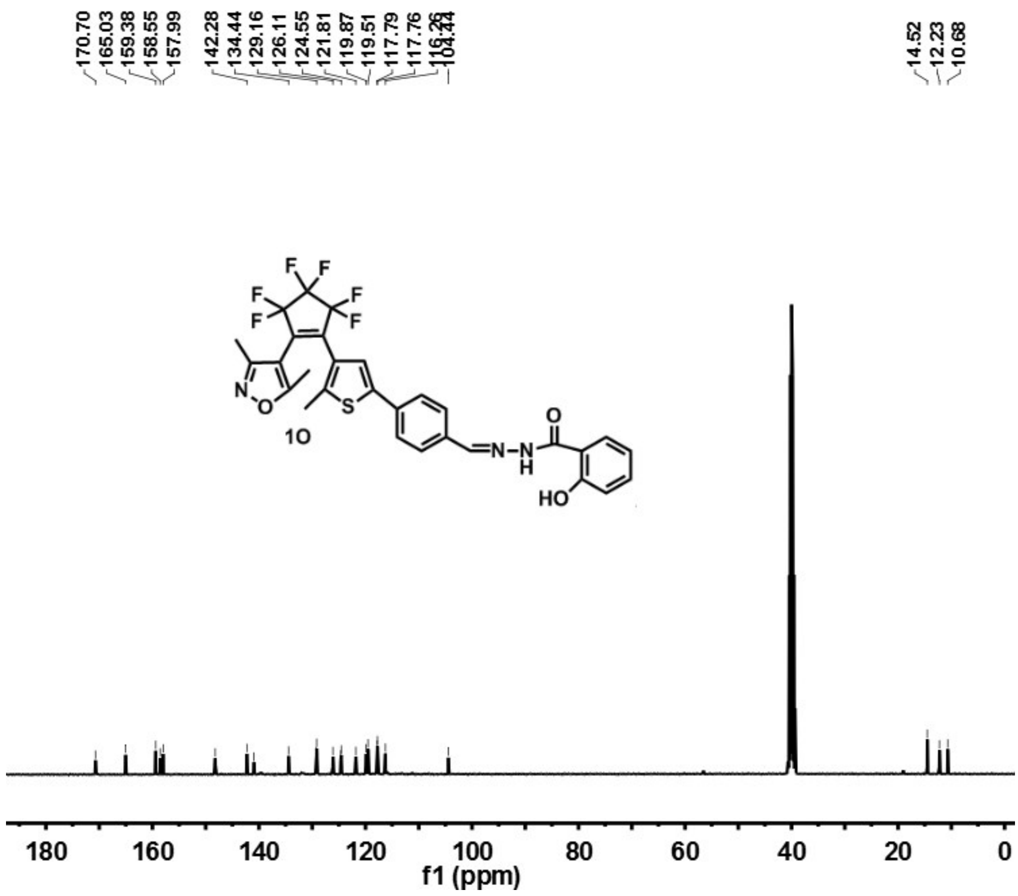

Figure S2. ^{13}C NMR spectrum of **1O**.

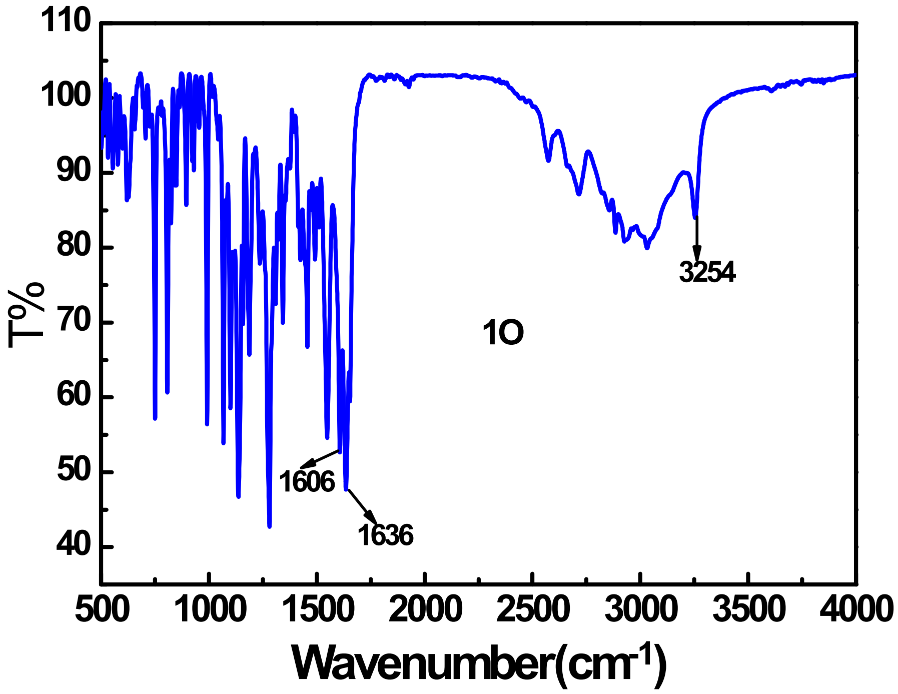

Figure S3. IR spectra of **1O**.

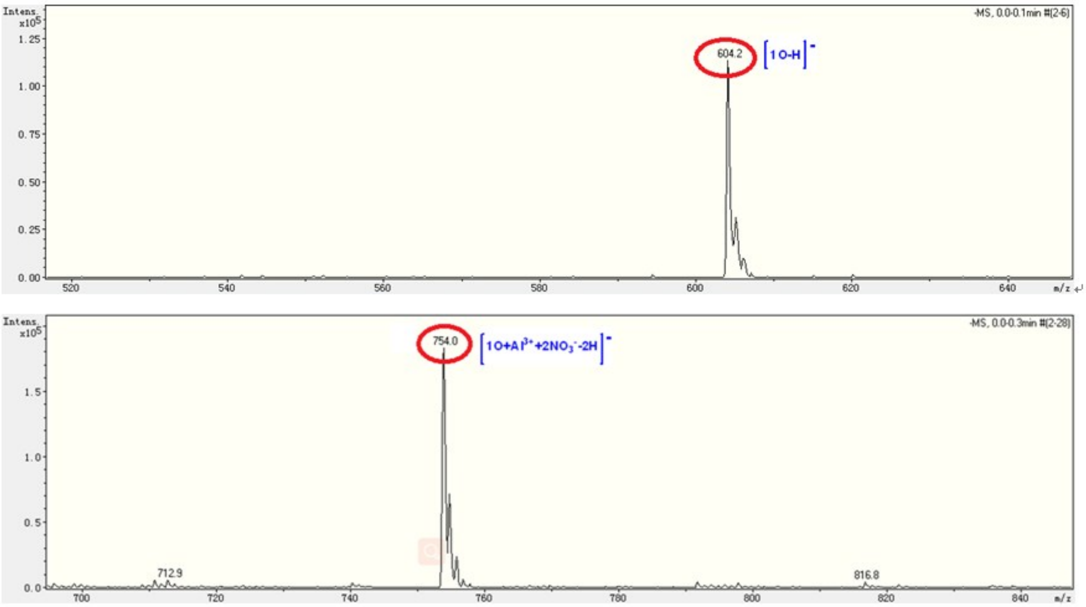

Figure S4. MS-ESI spectrum of **1O** and **1O**-Al^{3+}.

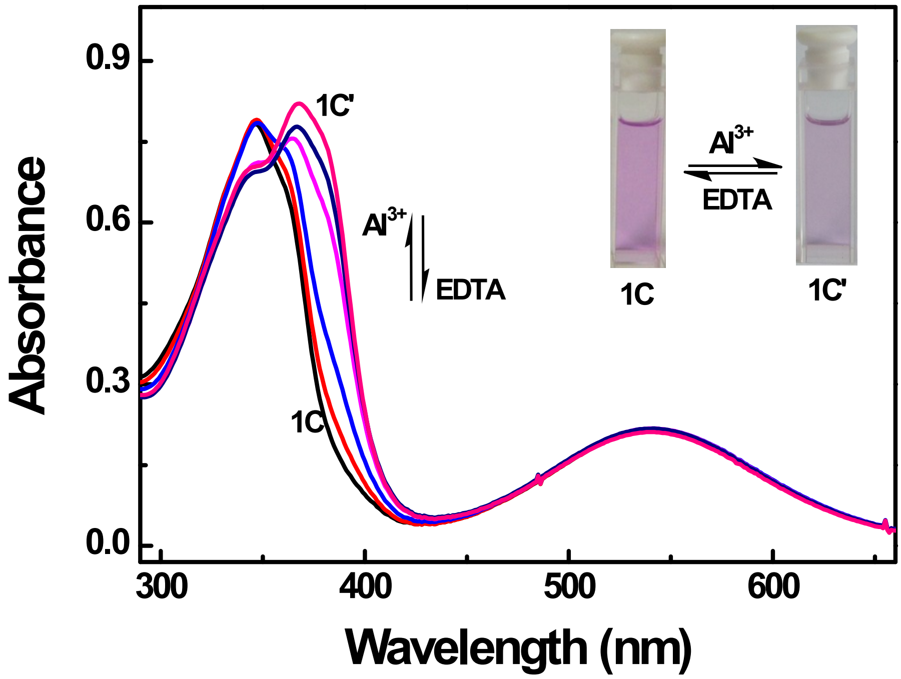

Figure S5. Absorption spectra changes of **1C** induced by Al^{3+}/EDTA in methanol solution (2.0×10^{-5} mol L^{-1}).

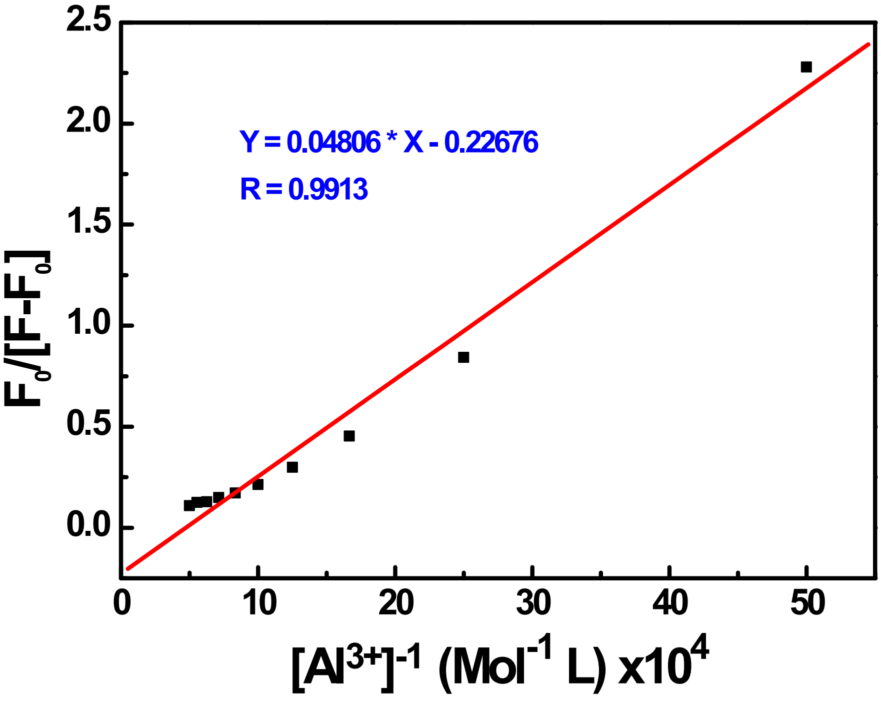

Figure S6. Hildebrand-Benesi plot based on the 1 : 1, the association constant of **1O** with Al^{3+} was calculated to be 4.72×10^4 L·mol^{-1}.

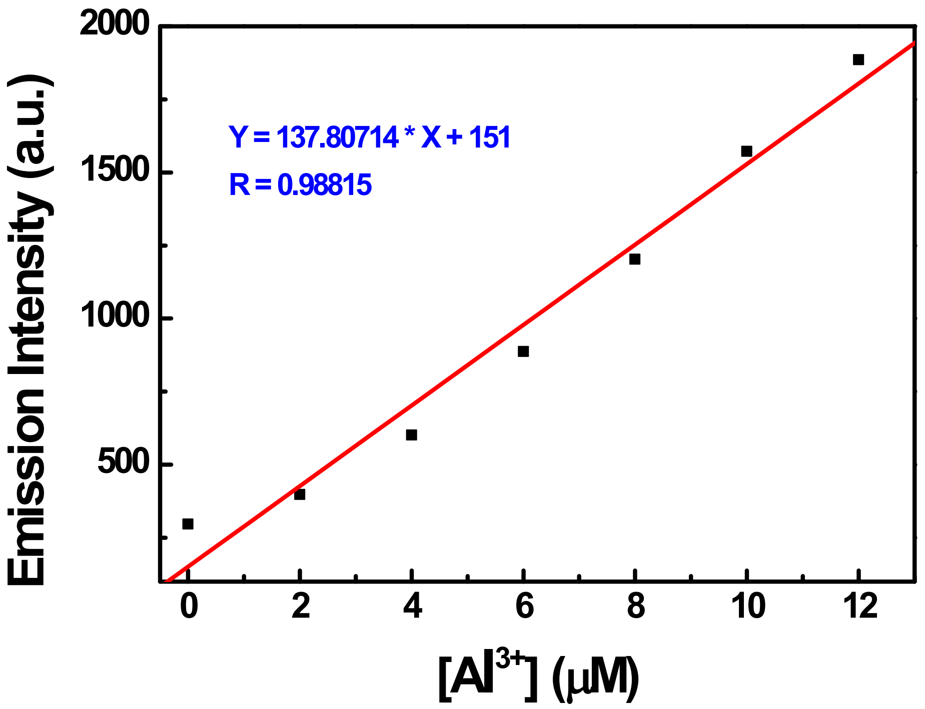

Figure S7. The limit of detection (LOD) for Al^{3+} was 1.24× 10^{-5} mol L^{-1}.

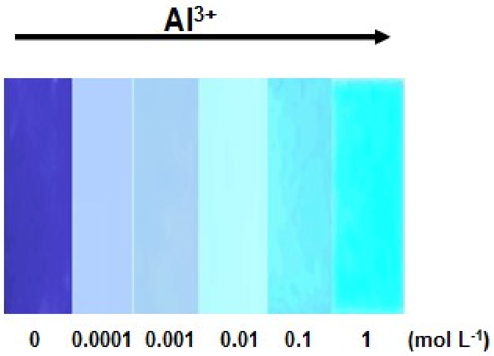

Figure S8. Fluorescence photos of Al^{3+} at different concentrations.
